# A nanohybrid synthesized by polymeric assembling Au(I)-peptide precursor for anti-wrinkle function

**DOI:** 10.3389/fbioe.2022.1087363

**Published:** 2022-12-12

**Authors:** Dan Liu, Yinong Huang, Jian Mao, Cheng Jiang, Lei Zheng, Qimei Wu, Hong Cai, Xiaojing Liu, Jingyao Dai

**Affiliations:** ^1^ Department of Talent Highland, The First Affiliated Hospital of Xi’an Jiaotong University, Xi’an, China; ^2^ Shaanxi Institute of Pediatric Diseases, Xi’an Children’s Hospital, Xi’an, China; ^3^ Graduate School of China Medical University, Shenyang, China; ^4^ Air Force Medical Center, Beijing, China; ^5^ Air Force Medical Center, Fourth Military Medical University, Xi’an, China

**Keywords:** anti-wrinkle, anti-aging, peptide, nanohybrid, biomaterial

## Abstract

A major sign of aging is wrinkles (dynamic lines and static lines) on the surface of the skin. In spite of Botulinum toxin’s favorable therapeutic effect today, there have been several reports of its toxicity and side effects. Therefore, the development of an effective and safe wrinkle-fighting compound is imperative. An antioxidant-wrinkle effect was demonstrated by the peptide that we developed and synthesized, termed Skin Peptide. Aiming at the intrinsic defects of the peptide such as hydrolysis and poor membrane penetration, we developed a general approach to transform the Skin Peptide targeting intracellular protein-protein interaction into a bioavailable peptide-gold spherical nano-hybrid, Skin Pcluster. As expected, the results revealed that Skin Pcluster reduced the content of acetylcholine released by neurons *in vitro*, and then inhibit neuromuscular signal transmission. Additionally, human experiments demonstrated a significant de-wrinkle effect. Moreover, Skin Pcluster is characterized by a reliable safety profile. Consequently, anti-wrinkle peptides and Skin Pcluster nanohybrids demonstrated innovative anti-wrinkle treatments and have significant potential applications.

## 1 Introduction

Due to the rapid pace of modern life, the aging population, and our changing lifestyles (staying up late, eating irregularly, etc.), fine wrinkles and loose skin have become the most prevalent symptoms of aging (Spada et al., 2019). This is one of the most obvious signs of aging skin that brings forth aging-related concerns (Ganceviciene et al., 2012). As a result of wrinkles, anxiety, depression, and psychological and physiological inferiority can occur, affecting both work and personal lives negatively (Tomas-Aragones and Marron, 2016). At present, Botulinum toxin (Botox), which inhibits muscle contractions by blocking acetylcholine, is the most widely used anti-aging modality (Nguyen et al., 2012; Tepper, 2020). However, Botox is expensive and has severe allergic reactions in humans, which cannot be ignored (Thanasarnaksorn et al., 2019). Therefore, the development of anti-wrinkle products is of great importance to address the issues of skin wrinkles.

There is an intrinsic factor that contributes significantly to the formation of wrinkles on the skin, which is the long-term muscle contraction by the facial muscles (Saluja and Fabi, 2017). By inhibiting the release of acetylcholine in nerve endings, neuromuscular signal transduction can be organized to paralyze muscles and reduce the formation of wrinkles. Rabphilin-3A (Rph3A) protein, which acts as a calcium-regulated nerve vesicle transporter on the inner membrane of nerve cells, can interact with SNAP25 to promote the release of acetylcholine. Blocking the interaction of Rph3A and SNAP25 can inhibit muscle fiber contraction and wrinkle formation (Nguyen et al., 2012; Ferrer-Orta et al., 2017; Stanic et al., 2017).

**FIGURE 1:**
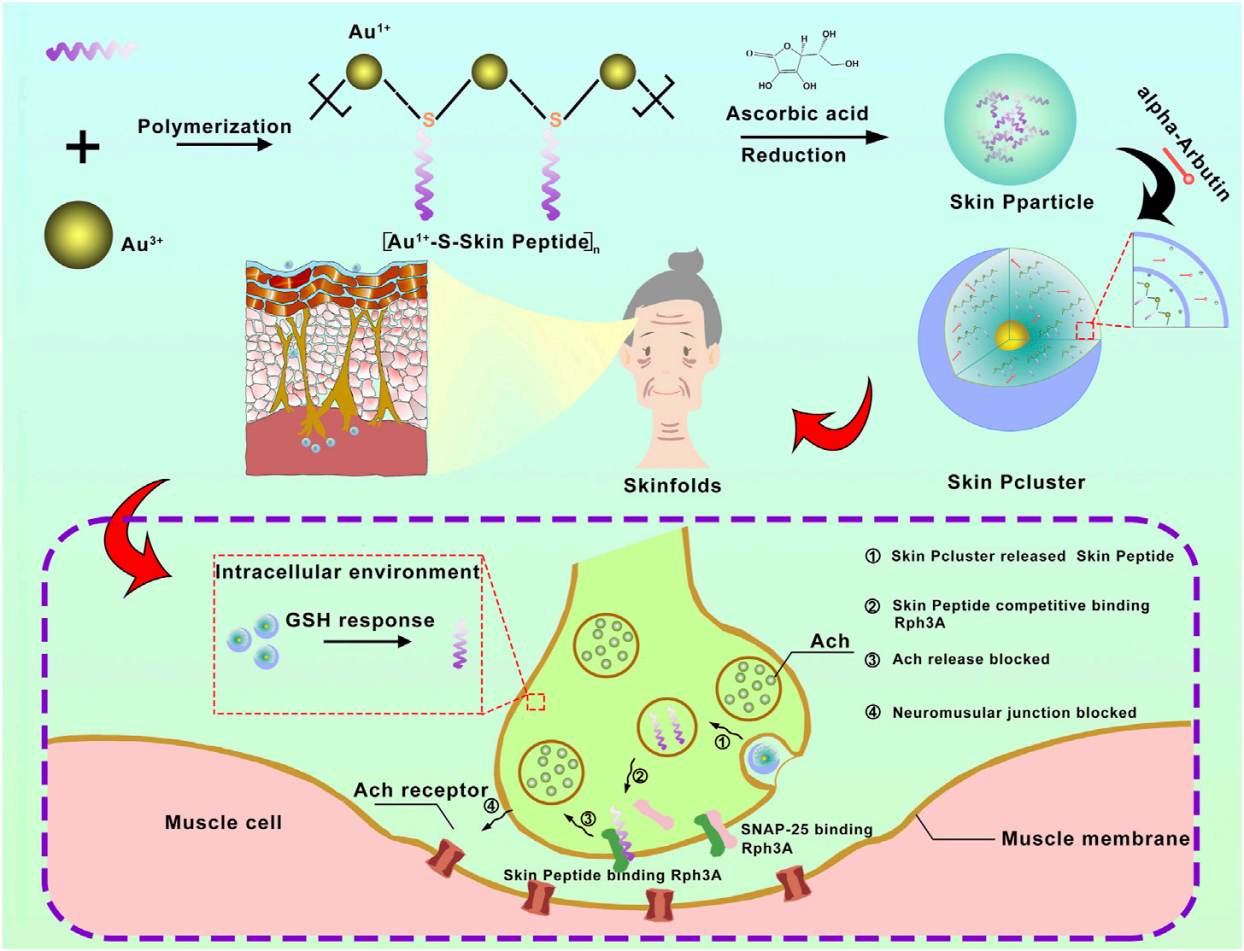
The schematic diagram for synthesis and biological function of Skin Pcluster. Firstly, a bionic peptide called Skin peptide was designed and synthesized. Then, the bioavailable peptide-gold spherical nano-hybrid, Skin Pcluster, was synthesized by self-assembled nanoengineering strategy. Skin Pcluster, similar to botulinum toxin and acetylhexapeptide-8, competitively binded Rph3A to inhibit the release of acetylcholine and then reduce wrinkles caused by excessive contraction of muscle.

There are numerous types of external cosmetics or injectable pharmaceuticals in which peptides are widely used as major anti-wrinkle ingredients both at home and abroad (Kennedy et al., 2020; Lim et al., 2020). The biomimetic peptide called Skin Peptide was designed and synthesized by an automated synthesizer in our study (Figure 1). Similar to Botox and acetyl hexapeptide-8, Skin Peptide played a role of anti-wrinkles by competitively inhibiting the interaction between SNAP25 and Rph3A. In chromaffin cells, this suppresses catecholamine release regulated by Ca^2+^, results in muscle to relax and paralysis, reduces the occurrence of dynamic lines and removes fine lines (Wang Y. et al., 2013; Multani et al., 2019a; Multani et al., 2019b). In addition, Skin Peptide has no risks of surgery, no restrictions on ages and no requirements for skin, which ensure a higher degree of safety than Botox. Due to inherent defects of peptide, such as low skin permeability, poor stability and rapid elimination, the clinical application of Skin Peptide is greatly limited (He et al., 2019a). To address these pharmacological barriers and improve clinical application of peptides, a growing number of optimizations for proteolytic resistance have been developed, including modifications and targeted delivery mechanisms (Lim et al., 2018). Although in optimizing peptide therapeutics, these two approaches have achieved some success, the translational applications of intracellular protein-protein interactions (PPI) remain a challenge (Katz et al., 2011).

As recent advances in nanotechnology, peptides are able to overcome inherent barriers more readily (Habibi et al., 2016; Li et al., 2017; Wei et al., 2017; Yuan et al., 2019). Through covalent or non-covalent modifications, the polypeptide has a stable structure with proteolytic resistance and cytomembrane permeability (Nevola and Giralt, 2015; Acar et al., 2017; Li et al., 2019). It has been demonstrated that peptide-derived nanomedicines, such as macromolecule-derived peptide nano micelles (Ying et al., 2016), peptide-coated nanoparticles (She et al., 2020), and peptide-based self-assembling nanostructures (He et al., 2019c; He et al., 2020), have attractive biological benefits including extended circulation times in the bloodstream, enhanced disease specificity and proteolytic stability (Yan et al., 2018; He et al., 2020; Zheng et al., 2021). The gold nanoparticle-conjugated peptides have the advantages of inherent inertia, low cytotoxicity and affordability, and have been more and more exploited and applied in clinical trials. In the past, AuNPs have been used as non-toxic vectors for the delivery of medicines and biomolecules. After conjugation, the complex structural characterizations of peptides (hydrophobicity, charge, and redox) are not conducive to the stable state of colloidal gold nanoparticles, resulting in subsequent aggregation and even sedimentation under the physiological conditions of increased ion concentration (Brinas et al., 2008). Thus, diminished colloidal stability usually results in prolonged release of therapeutic peptides, coupled with increased uptake by the reticuloendothelial system, ultimately leading to off-target toxicity and therapeutic failure (Ganceviciene et al., 2012).

In order to solve this problem, nanoengineering technology offers an attractive potential option (Yan et al., 2020a; Yan et al., 2021; Li et al., 2022). In mild conditions, the reducing agent transforms the peptide and HAucl_4_ into [Au(I)-S-peptide] _n_ and assembles the particles into a stable gold nanohybrid, a nanoscale gold sphere with a narrow distribution of gold particles (He et al., 2019b; Zheng et al., 2021; Wang et al., 2022). As one component of the drug delivery system, polypeptides significantly increased the loading efficiency. Further, it overcomes the inherent obstacles of peptides such as low skin permeability and poor stability.

## 2 Materials and methods

### 2.1 General remarks

All of these chemical reagents used in these experiments were obtained from Sigma-Aldrich unless otherwise indicated. All commercial products were directly used without further purification.

### 2.2 Synthesis of Skin Peptide

All peptides were prepared on a CSbio336X automated synthesizer using the technology of total chemical synthesis. After cleavage and deprotection in HF (Boc chemistry) or a mixture of reagents containing 88% TFA, 5% phenol, 5%H2O, and 2%TIPS (Fmoc chemistry), the crude products were precipitated with chilled diethyl and purified to homogeneity by preparative reversed-phase High-performance liquid chromatography (HPLC) on C18 columns. The molecular weights were identified by electrospray ionization mass spectrometry.

### 2.3 Preparation of Skin Pparticle and Skin Pcluster

The 0.5 ml HAuCl_4_ (10 mM) and 0.4 ml vitamin C ascorbic acid (0.2 M) were successively mixed with 4.5 ml of distilled water in a 50 ml beaker and stirred at a speed of 650 rpm for a few minutes. The above mixture solution gradually changed from colorless to wine red, indicating the formation of the solution of Au-nanoparticles. Subsequently, the 5.4 mg of peptide completely dissolved into the 21.6 ml distilled water under the ultrasonic condition. The solution of peptide was added into the solution of Au-nanoparticles and mixed evenly to obtain the solution of Skin Pparticle. Finally, the solution of Skin Pcluster was synthesized by adding 2% alpha-arbutin on the basis of solution of Skin Pparticle.

### 2.4 Physicochemical characterization of Skin Pparticle and Skin Pcluster

The morphology and dimensions were recorded on Transmission electron microscopy (TEM) (HT7700, 120 kV) at an acceleration voltage of 120 kV. These size distribution and zeta potential of nanoparticles were measured by dynamic light scattering (DLS). The surface chemical structure of nanoparticles was observed by Fourier transform infrared (FT-IR) spectroscopy (Nicolet 6700) and UV-vis spectroscopy (Shimadzu 3000).

### 2.5 GSH-triggered Skin Pcluster release Skin Peptide

Skin Pcluster and alpha-Arbutin standard were centrifuged at a speed of 12,000 rpm for 10 min, respectively. The release of alpha-Arbutin was qualitatively detected in the supernatant by UV-vis spectroscopy (Shimadzu 3000).

To illustrate the release of Skin Pcluster by GSH-triggered, the Skin Pcluster was dissolved in PBS buffer (pH 7.4) containing 10 mM or 10 μM glutathione (GSH) and incubated at 37°C for 1 h, 2 h, 3 h, 6 h and 12 h, respectively. Following this, the release of Skin Peptide in supernatants was quantified by HPLC. (Mobile phase A: 0.1% TFA/H_2_O, mobile phase B: 0.1% TFA/CH3CN, the flow rate of 1 ml/min, the gradient of 5–65%).

### 2.6 Study of acetylcholine release *in vitro*

The cultured neuron cells were induced by Skin Peptide and Botox respectively to detect the content of acetylcholine released in the supernatant. Untreated neuronal cell culture medium was used as negative control and Botox as positive control.

### 2.7 Study of paralytic relaxation of the gastrocnemius muscle in mice

The experiments involving animals were approved by the Xi’an Jiao tong University Ethics committee and followed the principles of the Laboratory Animal Center of Xi’an Jiao tong University.

C57BL/6 mice (aged 5–6 weeks) were randomly divided into the WT group (control group), Skin Pcluster group, Botox 0.004 U group, and Botox 0.008 U group, with no less than 3 mice in each group. The method of administration was intramuscular injection in the gastrocnemius muscle of mice with a dose of 20 μg per mouse (Cornet et al., 2020a; Cornet et al., 2020b). The images of the morphology were recorded by camera at 1 h and 2 h after injection. The effect of Skin Pcluster on gastrocnemius paralysis in mice was measured with force and drop distance by a digital force gauge.

### 2.8 Anti-wrinkle effect studies on humans

The experiments involving human were approved by the Air Force Medical University Ethics committee. Healthy subjects were recruited and randomly assigned to the group with/without anti-wrinkles treatment. The eye cream, face cream, essence and mask containing 10% Skin Pcluster were used on the skin of eyes, face, hands and neck respectively. The eye cream, face cream and essence were used twice a day and mask were used twice a week for 4 weeks. Pre- and post-treatment images were acquired by VISIA.

### 2.9 Statistics

The experimental results were compared between the two groups of data by independent sample *t*-test. One-way ANOVA and Tukey post-analysis, or logarithmic rank test was used to compare more than three groups (*p < 0.05, **p < 0.01, and ***p < 0.001).

## 3 Results

### 3.1 Design and synthesis of Skin Peptide

**FIGURE 2:**
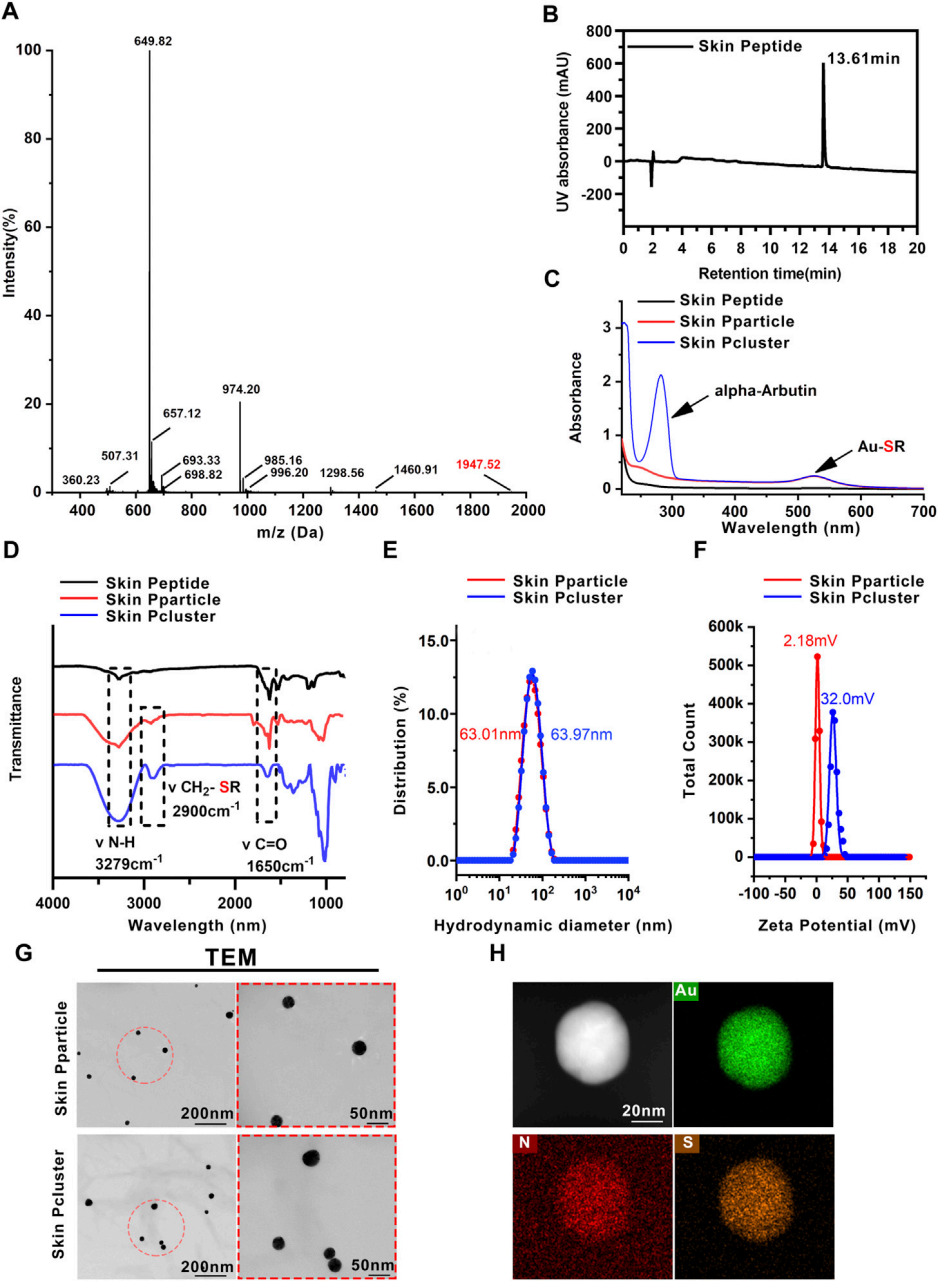
Characterization of Skin Pcluster. **(A)** The molecular weight of the Skin Peptide was identified by electrospray ionization mass spectrometry. **(B)** Retention time and purity of Skin Peptide was quantitatively analyzed by high performance liquid chromatography (HPLC) on a C18 column at 214 nm. **(C)** UV-vis spectra of Skin Peptide, Skin Pparticle and Skin Pcluster. The characteristic absorption peaks of Au-SR and alpha-Arbutin (aromatic compound) were visualized at 520 nm and 283nm, respectively. **(D)** FTIR spectra of the Skin Peptide, Skin Pparticle and Skin Pcluster were determined after lyophilization. Three absorption peaks at 3279 cm^−1^, 2900 cm^−1^ and 1650 cm^−1^ demonstrated the stretching vibration of N-H, CH_2_-SR, and C=O, respectively. **(E)** Hydrodynamic diameter distributions of the Skin Pparticle and Skin Pcluster were performed by dynamic light scattering. **(F)** The surface charge (Zeta potential) of the Skin Pparticle and Skin Pcluster was measured in PBS at pH7.4. **(G)** TEM images of the Skin Pparticle and Skin Pcluster were obtained by a CCD camera. The red circles represent magnification-related regions of the Skin Pparticle and Skin Pcluster. **(H)** Elemental analysis of Skin Pcluster was recorded by HRTEM.

According to the report, Rph3A is a calcium-regulated neural vesicle transporter on the inner membrane of nerve cells, which interacts with SNAP25 to block the release of intracellular acetylcholine, and then inhibit the contraction of muscle and achieve the effect of anti-wrinkle (Lee et al., 2008). Hence, we focused on Rph3A as the target protein (PDB code: 5LOW) and synthesized Skin Peptide with the help of total chemical synthesis technology utilizing native chemical ligation. Subsequently, the relative molecular weight of the peptide was determined to be 1947 Da by mass spectrometry (Figure 2A), which further confirmed that the sequence of the synthesized peptide was consistent with our design, namely the Skin Peptide. The purity of the Skin Peptide was determined by HPLC. As shown in Figure 2B, the synthesized Skin Peptide showed a single chromatographic peak with a 13.61 min retention time. These results illustrated that Skin Peptide was successfully synthesized.

### 3.2 Synthesis and characterization of Skin Pcluster

According to the previous report (Yan et al., 2020b), the preparation of Skin Pcluster was conducted under mild conditions by a three-step “one-pot” reaction. Specifically speaking, in step 1) the reduction of the flavescent Au^3+^ by mercaptan in Skin Peptide-SH forms a polymerized structure of [Au (I)-SH-Skin Peptide] n, termed (Au^1+^-S-Skin Peptide); step 2), subsequently, (Au^1+^-S-Skin Peptide) is reduced to polymerized Au-peptide nanoparticles Skin Peptide Nanoparticles, termed Skin Pparticle. Finally, to endow nanoparticles with more biological properties and improve the physical stability of Skin Pparticle, in step 3), add alpha-Arbutin to trigger the Skin Pparticle into the Skin Pcluster in an electrostatic absorption manner (Figure 1).

In the first step, (Au^1+^-S-Skin Peptide) was formed by spontaneous coordination between Au^3+^ in HAucl_4_ and sulfated Skin Peptide. As shown in Figures 2C, D, the disappearance of the characteristic absorption peak of sulfhydryl groups in Skin Peptide-SH and the appearance of Au-SH absorption peak in Skin Pparticle and Skin Pcluster were confirmed by UV-Vis and FT-IR spectroscopy, which indicated successful synthesis of the (Au^1+^-S-Skin Peptide).

In the second step, the solution containing Vitamin C-ascorbic acid was reduced to the Skin Pparticle, and then was changed from colorless to wine-red to form the Skin Pparticle. The characteristic absorption peaks of peptides were found in the FT-IR spectrum (Figure 2D). In addition, as illustrated in Figure 2G, TEM images of the Skin Pparticle showed that the average size and shape distribution of particles was homogeneous at about 30 nm. It was worth noting that, in Figure 2E, the sizes of Skin Pparticle measured by dynamic light scattering (DLS) were 63.01 nm larger than that observed by TEM, which was attributed to the fact tant particles are easily stretched in solution.

In the third step, alpha-Arbutin was added into the two-step solution inducing the Skin Pparticles to form a more stable Skin Pcluster in a manner of electrostatic assembly. As shown in Figure 2C, the characteristic absorption peak of alpha-Arbutin was observed at 283 nm. With the addition of alpha-Arbutin, the zeta potential was gradually increased from 2.18 mV to 32.0 mV, indicating that the Skin Pcluster had excellent colloidal stability (Figure 2F). Additionally, the size distribution of the Skin Pcluster was homogenous and at approximately 30–40 nm observed by TEM (Figure 2G). The element overlay and TEM images presented the uniform distribution of nitrogen (N), sulfur (S), and gold (Au) in the Skin Pcluster indicating the good uniformity of Skin Pcluster (Figure 2H). The elements constituent of Skin Pcluster were consistent with the constituents of HAuCl_4_, Skin Peptide, alpha-Arbutin, and Vitamin C-ascorbic acid by Energy dispersive X-ray spectroscopy (EDS) analysis (Supplementary Figure S1). Taken together, these results demonstrated that Skin Pclusters were successfully prepared and were homogeneous spherical gold-peptide nanohybrids.

### 3.3 Skin Pcluster can respond to GSH-triggered to release Skin Peptide

**FIGURE 3:**
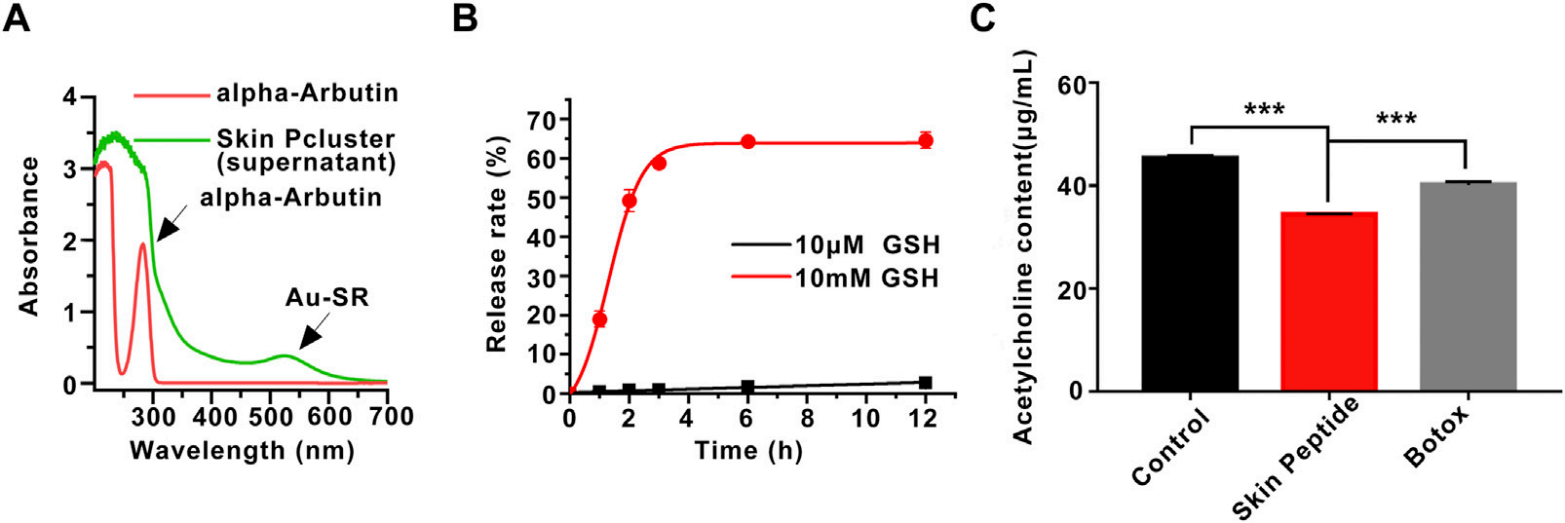
Stability of Skin Pcluster and the release of acetylcholine induced by Skin Peptide. **(A)** The release of alpha-Arbutin from Skin Pcluster in the supernatant was determined by UV-vis spectroscopy. **(B)** The release of Skin Peptide was detected in PBS containing 10 μM or 10 mM GSH at different times. **(C)** The measurement of acetylcholine in the supernatant. Data were shown as mean ± sem (n = 3). *P* values were obtained by *t*-test.

To endow nanoparticles with more biological properties and improve their physical stability, alpha-arbutin was added to induce the assembly of Skin Pparticle to form Skin Pcluster by electrostatic adsorption (Shin et al., 2019). The alpha-Arbutin can be qualitatively released in the supernatant of the Skin Pcluster. As demonstrated in Figure 3A, compared with the standard of alpha-arbutin, there was no characteristic absorption peak of alpha-arbutin at 283 nm in the supernatant of Skin Pcluster, indicating that the alpha-arbutin in Skin Pcluster was easily released. Additionally, the characteristic absorption peak of Au-SR can still be observed in the supernatant of Skin Pcluster, which is consistent with the result described above that Au-S bond was relatively stable.

The therapeutic effect of Skin Pcluster targeting intracellular PPI is related to the effective concentration of Skin Peptide in the cytoplasm (Yan et al., 2020a). Therefore, another essential function of the Skin Pcluster is the efficient release of functional Skin Peptide into the targeted cells. According to the literature (He et al., 2020), the Au-S bond is a robust chemical linkage in extracellular physiological environment, and it can be disrupted by a high concentration of thiol. GSH, as a general non-protein mercaptan in organisms, was discovered in intracellular at a concentration of millimoles and extracellular at a concentration of micromoles (Wang X. et al., 2013). To further simulate the high concentration of GSH could stimulate the quantitative release of Skin Peptide from the Skin Pcluster, Skin Peptide was monitored by analytical high-performance liquid chromatography (HPLC) at different time points.

As shown in Figure 3B, Skin Pcluster maintained basically intact after incubation in the PBS containing 10 μM GSH (pH7.4) for 12h, and release of Skin Peptides were much lower than 10%. In stark contrast, the release of Skin Peptides had reached 20% after Skin Pcluster incubated with 10 mM GSH for 1 h. Meanwhile, with the extension of incubation time, the release of Skin Peptides gradually increased. When Skin Pcluster was incubated for 12 h, the release of Skin Peptides reached 65%. These results illustrated that Skin Peptide can be released under high GSH conditions.

### 3.4 *In vitro* inhibition of acetylcholine release

Previous findings have indicated that Skin Peptide can be released in response to GSH. Based on this, we further investigated whether the released Skin Peptide could affect the content of the neurotransmitter acetylcholine. It has been reported that Botox can inhibit the release of acetylcholine, and thus Botox was used as a positive control. As shown in Figure 3C, Skin Peptide remarkably inhibited the released acetylcholine compared with the Control group, which was consistent with our previous speculation. Excitingly, in contrast to the Botox group, Skin Peptide significantly suppressed the release of acetylcholine. Collectively, these results demonstrated that Skin Peptide could decrease the release of the neurotransmitter acetylcholine.

### 3.5 Study of paralytic relaxation of the gastrocnemius muscle in mice

**FIGURE 4:**
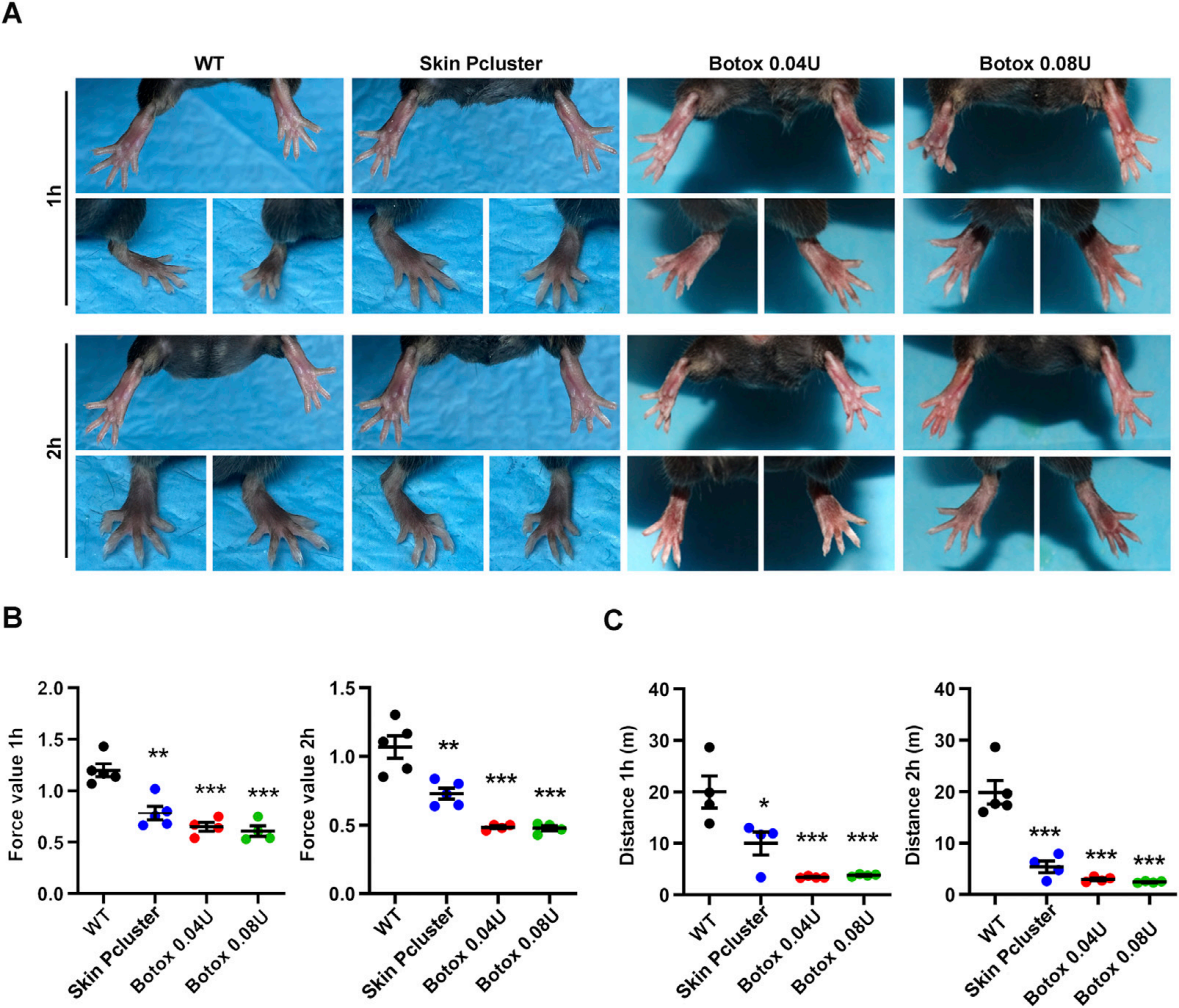
Study on paralytic relaxation of gastrocnemius in C57BL/6 mice. **(A)** The photographs of feet were recorded at 1h and 2 h after injection. **(B**,**C)** The effects of Skin Pcluster on gastrocnemius paralysis in mice were measured by force and drop distance with a digital force gauge. Statistic data were represented as mean ± sem (n = 4 or 5/group). *P* values were obtained by *t*-test.

On previous study, the Skin Pcluster could inhibit the release of the neurotransmitter acetylcholine. To further confirm whether the Skin Pcluster had secondary effects on muscle movement in mice, such as muscle expansion and relaxation. The healthy mice were randomly divided into four groups, and were injected intramuscular with PBS(WT), Skin Pcluster (1 mg/kg), Botox (0.04U/Kg) or Botox (0.08U/Kg) at different time points, respectively. As shown in Figure 4A, compared with WT group, toes gradually converged and gastrocnemius relaxed in the hindlimbs of Skin Pcluster-treated mice after 1 h injection. Additionally, there was no significant difference in muscle relaxation between Botox (0.08 U/Kg, 0.04 U/Kg) and Skin Pcluster (1 mg/kg). Two hours after injection, the hindlimbs of the mice with Skin Pcluster-treatment displayed visible gastrocnemius weakness and toe aggregation.

Then, the test of mouse grasping power and the measurement of running distance were further used to quantitatively evaluate the muscle relaxation degree of the hindlimbs of mice after administration. As shown in Figure 4B, compared with WT group, the grasping power of Skin Pcluster group was significantly decreased 1h and 2 h after injection, respectively. Meanwhile, the grasping force values of Botox positive control group (0.04 U/Kg and 0.08 U/Kg) were also lower than those of WT group, indicating that the quantitative evaluation of the degree of muscle relaxation in the hindlimbs of mice was feasible. As expected, after 1h and 2 h treatment, the running distance of Skin Pcluster group was significantly less than the WT group, which was consistent with the results of the grasping force (Figure 4C). In conclusion, these results suggested that the Skin Pcluster, as a novel self-assembling nanomedicine, produced a relaxant effect on the movement of gastrocnemius muscle.

### 3.6 Anti-wrinkles evaluation in humans

**FIGURE 5:**
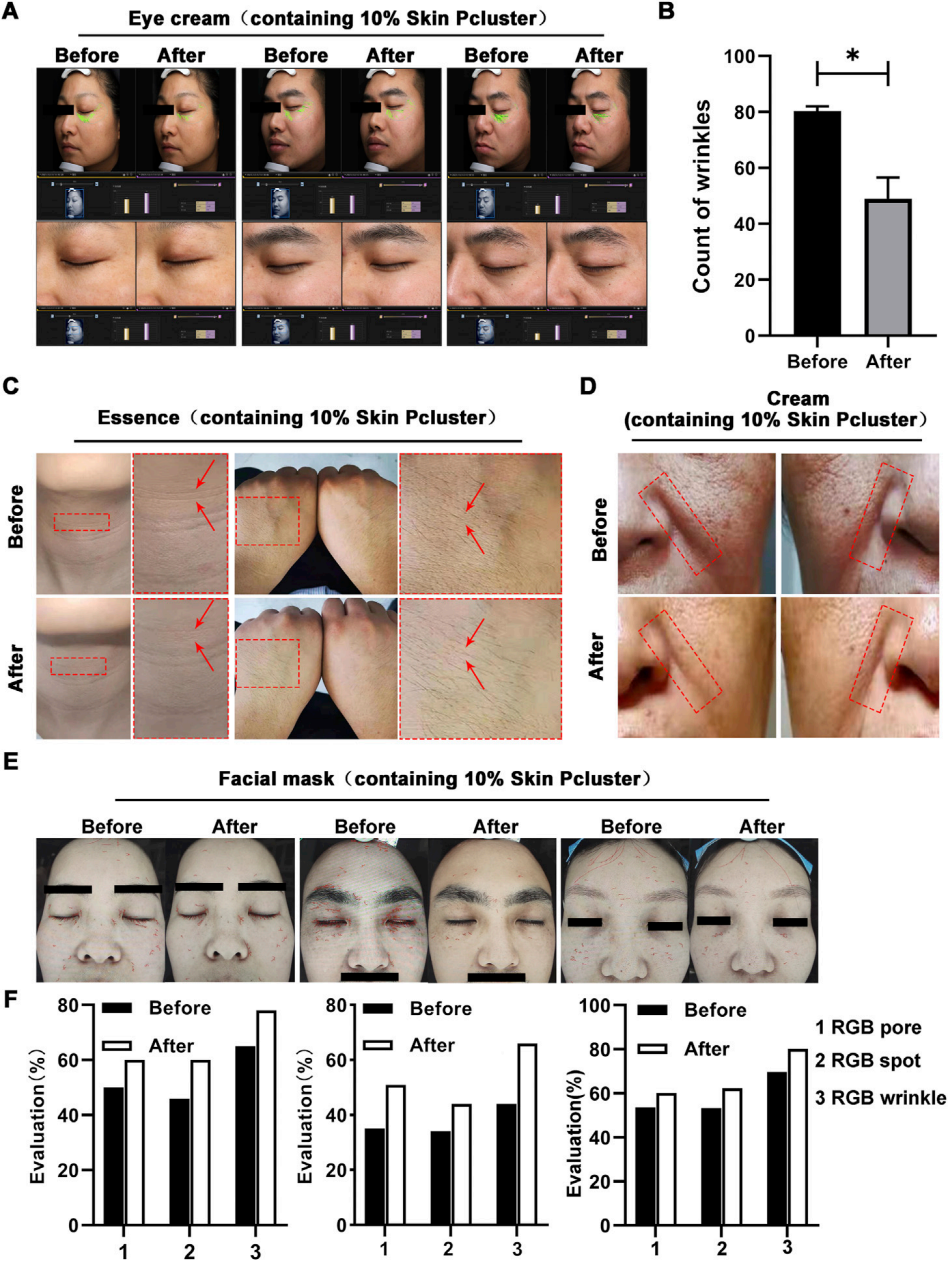
Anti-wrinkle effect of Skin Pcluster in humans. **(A**,**B)** Before and after the treatment with eye cream (including 10% Skin Pcluster), VISIA was used to visualize the wrinkles around the eyes of the recruited volunteers. The number of wrinkles was quantified. **(C)** The representative photographs of healthy subjects’ neck and hand before and after treatment with essence containing 10% Skin Pcluster. **(D)** Images of facial wrinkles treated with a cream containing 10% Skin Pcluster. **(E**,**F)** Images of wrinkles around the eyes and on the forehead of the recruited volunteers were obtained by VISIA before and after treatment with eye cream containing 10% Skin Pcluster. p values were obtained by *t*-test.

Previous studies have revealed that injection with Skin Pcluster can exert an inhibitory effect on the release of the neurotransmitter acetylcholine, which in turn blocks neuromuscular signaling. We further examined whether the suppression of neuromuscular transmission signaling can reduce or eliminate wrinkles caused by prolonged contractions of muscle. As expected, a significant reduction in eye wrinkle texture (marked in green) was observed in three subjects after treatment with eye cream containing 10% Skin Pcluster (Figure 5A). Using the VISIA Skin analysis system to further quantify the results of eye wrinkle improvement, it was found that when subjects applied an eye cream containing 10% Skin Pcluster, the number of wrinkle textures was reduced by half and the crow’s feet texture covering the eye area was significantly improved (Figure 5B). Furthermore, none of the subjects experienced significant discomfort or allergic reactions after using the eye cream containing 10% Skin Pcluster.

To further confirm the reliability of Skin Pcluster’s anti-wrinkle effect, essence containing 10% Skin Pcluster was applied to subjects’ neck and hands respectively. As expected, the wrinkle texture of neck became lighter by visual inspection (marked by red arrows) when the neck was treated with essence containing 10% Skin Pcluster compared to the skin before application essence. Meanwhile, it was observed that in the hand, skin pores narrowed and skin tightened after application of essence (Figure 5C). In addition, there was no obvious discomfort or allergic reaction for all subjects after using the essence containing 10% Skin Pcluster. Next, the subjects with deep nasolabial folds were selected to receive a cream containing the 10% Skin Pcluster. Comparing the face area marked by the red dashed box, the nasolabial folds gradually became lighter after usage of the cream (Figure 5D).

Finally, VISIA skin analysis system was used to detect wrinkles across the face. It was consistently found that in subjects who applied mask with 10% Skin Pcluster, not only the number of wrinkles (red) was reduced, but also the pores and pigmentation of the skin were improved (Figures 5E, F). Moreover, in the analysis system, the higher value of RGB spots, RGB pores and RGB texture, the better the skin condition is. And it could be seen that these three indicators increased after the use of the mask. Furthermore, all the above subjects had no obvious discomfort or allergic reaction after using the mask. Taken together, the above results illustrated that the Skin Pcluster had an obvious anti-wrinkle effect. Notably, even intraperitoneal administration of Skin Pcluster did not result in loss of body weight, changes in H&E staining of organs and the function of liver and kidney (Supplementary Figure S2), suggesting the safety of the Skin Pcluster.

## 4 Discussion

Wrinkles (dynamic texture and static wrinkles) originate from the excessive contraction of muscle fibers, and the key to reduce wrinkles is to block the excessive contraction of muscle fibers. Rph3A, a calcium-regulated vesicle transporter located on the cell membrane of neurons, is involved in the release of neurotransmitters by interacting with SNAP25 (Ferrer-Orta et al., 2017). Therefore, we first designed and synthesized a bionic peptide that competitively binds to Rph3A for anti-wrinkle. However, due to the inherent defects of peptide (easy hydrolysis, poor membrane permeability, etc.), the further development and clinical application of it are limited. Here, we have developed a general nano-engineering technology to convert biomimetic peptides into stable and bioavailable peptide-gold spherical nanohybrids, called Skin Pcluster, under simple and mild chemical reaction conditions. According to previous TEM images, the Skin Pcluster had a particle size distribution of 30–40 nm and easily penetrated the skin. Additionally, Skin Peptide was covalently bound with Au^1+^ and was not easily degraded by the proteasome in skin. When the Skin Pcluster entered the neurons on muscle layer, Skin Peptides could be released in response to the intracellular higher GSH, competitively binds to Rph3A. Furthermore, the interaction of SNAP25-Rph3A was inhibited and the release of neurotransmitter acetylcholine was reduced, thus inhibiting excessive contraction of muscle to achieve anti-wrinkle effects. Through nano-engineering modification, Skin Pcluster was enhanced on the performance of penetrating skin, which can be applied on the surface of skin to play the role of anti-wrinkle. Compared with invasive operation of intramuscular injection, Skin Pcluster is more convenient, comfortable and safe to apply on the surface of skin, which further expands the possibility of widespread application of Skin Pcluster in the market in the future.

In this study, with the aid of computer-aided drug design, a biomimetic peptide was designed to target the interaction between intracellular proteins and proteins (Yu and MacKerell, 2017; Battini et al., 2021). There is no doubt that peptide-based nanostructures have increased exponentially over the past few decades, which is attributed to their bio-inspired properties. Biomimetic strategies have been widely used in various types of research, such as tumor therapy (He et al., 2022; Peng et al., 2022; Wu et al., 2022), vaccination (Peng et al., 2022) and wound dress (Mirhaj et al., 2022). And it has been reported that nanocarriers with bionic cell membranes were an effective and safe delivery system for osteosarcoma therapy (Huang and Kiick, 2022). The shell of ZIF-8 has multiple functions to develop as a safe and effective platform for novel spore-based vaccine. In addition, the biomimetic strategies were applied to the preparation of bi-layer chitosan wound dressings. Generally speaking, the bionic strategy opens the way for a more systematic study of protein-protein interaction or nanoscale, which may eventually lead to the development of rationally designed engineered nanoparticles for biosynthesis. In the further continuation, our team will utilize bionic strategies to focus on the development of a new generation of collagen technology.

## Data Availability

The original contributions presented in the study are included in the article/Supplementary Material, further inquiries can be directed to the corresponding authors.
