# Supplementary material for: A nanohybrid synthesized by polymeric assembling Au(I)-peptide precursor for anti-wrinkle function
Source: Front Bioeng Biotechnol. 2022 Dec 12;10:1087363. doi: 10.3389/fbioe.2022.1087363 (PMC9790933; doi:10.3389/fbioe.2022.1087363)
Supplement: Supplementary file 1 [file DataSheet1.docx]

Supplementary Material

# 1. Supplementary Figure

#
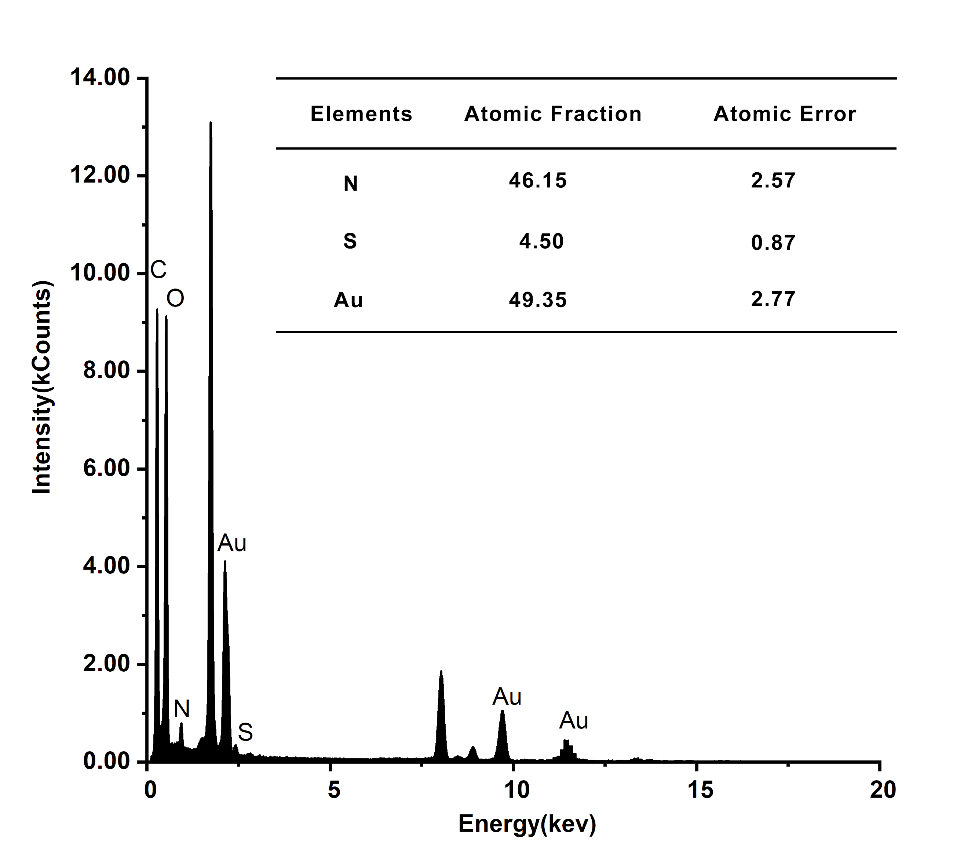


# Supplementary Figure 1. EDS quantitative element analysis of Skin Pcluster.


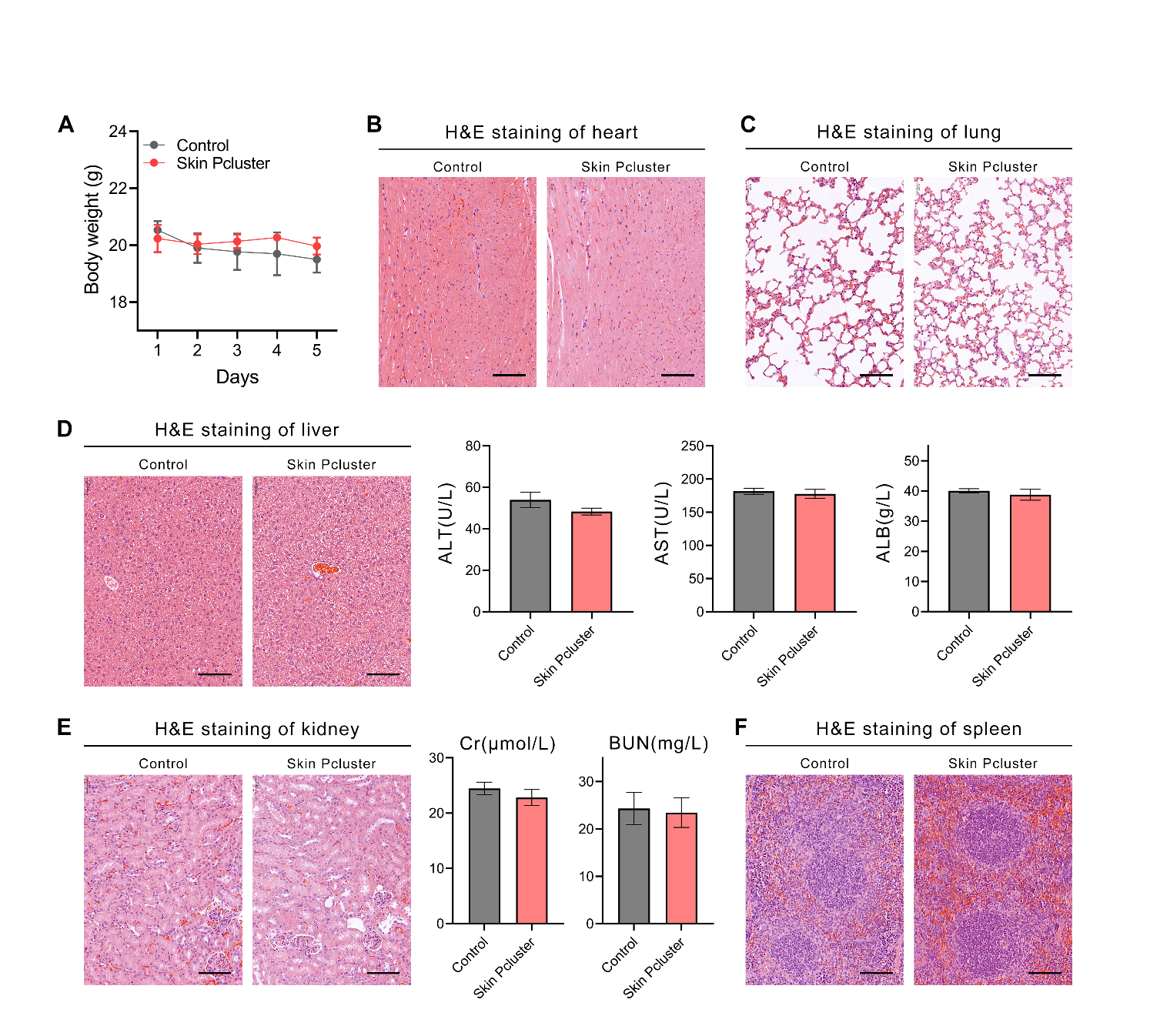


**Supplementary Figure 2. Safety assessment of Skin Pcluster. (A)** Changes in body weight of C57BL/6 mice during administration. **(B-C).** H&E staining of heart (B) and lung sections (C). **(D)** The effects of Skin Pcluster on the liver were shown in images of H&E staining and serum indicators of liver, including alanine aminotransferase (ALT), aspartate transaminase (AST) and serum albumin (ALB). **(E)** H&E staining of renal pathological sections and creatinine (CR), blood urea nitrogen (BUN) in serum were tested in respond to function of kidney. **(F)** Representative images of H&E staining of spleen. Scale bar, 100 µm. The data were represented as mean ± sem (n=3/group).

# 2. Supplementary Methods

## Safety evaluation of Skin Pcluster

C57BL/6 mice were randomly divided into control group and Skin Pcluster group, and were injected PBS(Control)/Skin Pcluster intraperitoneally at the dose of 1mg/kg for 5 days. After administration, the mice were euthanized and the hearts, liver, spleen, lungs and kidneys were isolated from the mice. Hematoxylin and eosin (H&E) staining was performed on the tissue after fixation in 4% paraformaldehyde, embedding and section. Serum aspartate transaminase (ALT), alanine aminotransferase (AST), creatinine (CR) and blood urea nitrogen (BUN) of serum were measured by quantitative enzyme-linked immunosorbent assay (ELISA) kits according to the manufacturer’s instructions.
